# Redefining Remission Induction Chemotherapy Ineligibility by Early Mortality in De Novo Acute Myeloid Leukemia

**DOI:** 10.3390/jcm10245768

**Published:** 2021-12-09

**Authors:** You-Cheng Li, Yu-Hsuan Shih, Tsung-Chih Chen, Jyh-Pyng Gau, Yu-Chen Su, Mei-Hui Chen, Chiann-Yi Hsu, Cai-Sian Liao, Chieh-Lin Jerry Teng

**Affiliations:** 1 Division of Hematology/Medical Oncology, Department of Medicine, Taichung Veterans General Hospital, Taichung 40705, Taiwan; johnatelee@gmail.com (Y.-C.L.); rollingstone07@gmail.com (Y.-H.S.); tjchen9477@gmail.com (T.-C.C.); jennysunm@hotmail.com (Y.-C.S.); meihui12060819@gmail.com (M.-H.C.); 2 Graduate Institute of Clinical Medicine, College of Medicine, National Taiwan University, Taipei 10617, Taiwan; 3 Division of Hematology and Oncology, Department of Medicine, Taipei Veterans General Hospital, Taipei 40705, Taiwan; jpgau@vghtpe.gov.tw; 4 School of Medicine, National Yang Ming Chiao Tung University, Taipei 112201, Taiwan; 5 Department of Nursing, Taichung Veterans General Hospital, Taichung 40705, Taiwan; 6 Biostatistics Task Force, Taichung Veterans General Hospital, Taichung 40705, Taiwan; chiann@vghtc.gov.tw; 7 Department of Medical Research, Taichung Veterans General Hospital, Taichung 40705, Taiwan; enelya2323@gmail.com; 8 Department of Life Science, Tunghai University, Taichung 407224, Taiwan; 9 School of Medicine, Chung Shan Medical University, Taichung 40201, Taiwan; 10 College of Medicine, National Chung Hsing University, Taichung 40227, Taiwan

**Keywords:** AML, early mortality, age, performance status, LDH

## Abstract

The therapeutic strategies for acute myeloid leukemia (AML) patients ineligible for remission induction chemotherapy have been improving in the past decade. Therefore, it is important to define ineligibility for remission induction chemotherapy. We retrospectively assessed 153 consecutive adult de novo AML patients undergoing remission induction chemotherapy and defined early mortality as death within the first 60 days of treatment. The 153 patients were stratified into the early mortality group (n = 29) and the non-early mortality group (n = 124). We identified potential factors to which early mortality could be attributed, investigated the cumulative incidence of early mortality for each aspect, and quantified the elements. The early mortality rate in our study cohort was 19.0%. Age ≥ 65 years (odds ratio (OR): 3.15; 95% confidence interval (CI): 1.05–9.44; *p =* 0.041), Eastern Cooperative Oncology Group performance status ≥ 2 (OR: 4.87; 95% CI: 1.77–13.41; *p =* 0.002), and lactate dehydrogenase ≥ 1000 IU/L (OR: 4.20; 95% CI: 1.57–11.23; *p =* 0.004) were the risk factors that substantially increased early mortality in AML patients. Patients with two risk factors had a significantly higher early mortality rate than those with one risk factor (68.8% vs. 20.0%; *p* < 0.001) or no risk factors (68.8% vs. 9.2%; *p* < 0.001). In conclusion, older age, poor clinical performance, and a high tumor burden were risks for early mortality in AML patients receiving remission induction chemotherapy. Patients harboring at least two of these three factors should be more carefully assessed for remission induction chemotherapy.

## 1. Introduction

The incidence of 1–2 cases per 100,000 people makes acute myeloid leukemia (AML) the most common type of leukemia in adults [1]. Although the precise pathogenesis of AML remains inconclusive, the abnormal proliferation and differentiation of hematopoietic stem cells are considered to result in the development of AML. The World Health Organization has proposed that the diagnosis of AML be confirmed if ≥20% of the nucleated cells from either the peripheral blood or the bone marrow are myeloblasts [2]. Remission induction chemotherapy is the first step in the treatment of AML. Patients with high-risk cytogenetics or genetic mutations may need allogeneic hematopoietic stem cell transplantation to improve their survival [3].

In the past, “7 + 3” or “7 + 3”-like regimen induction with the intention to cure may have been the only option for newly diagnosed AML patients. While the outcome for patients who were ineligible for the “7 + 3” remission induction used to be dismal, therapeutic strategies for these patients have shown an improvement in the past decade, such as the venetoclax-based regimen [4]. A consequence of these therapeutic improvements is that the definition of chemotherapy ineligibility has become an important issue [5]. However, the criteria for identifying patients who are ineligible for remission induction chemotherapy have not yet been standardized. Significant comorbidities [6] or a high probability of induction failure [7] may be the primary reasons for ineligibility for remission induction chemotherapy. Among all the potential approaches to validate the criteria of ineligibility for remission induction chemotherapy, early death is an easy and feasible one.

Various etiologies may result in early mortality in AML patients. Among all the risks determining early mortality in AML patients, older age is the primary one because older patients usually have a worse performance status and more comorbidities than young patients [8]. Besides, from molecular [9] and cytogenetic [10] perspectives, older patients are more likely to have biologically poor-risk AML. While low performance and a higher tumor burden have also been attributed to early mortality in AML treatment [11], and while the Ferrara unfitness criteria further provide a precise prediction tool for shorter-term mortality in AML patients after intensive chemotherapy [12], the incorporation of these factors in the rapid prediction of early mortality due to AML remains unclear, and further investigation is required.

We conducted this retrospective study to identify the risk factors that can be attributed to early mortality in de novo AML patients undergoing remission induction chemotherapy. Furthermore, this study aimed to incorporate the identified risks in redefining remission induction chemotherapy ineligibility using early mortality along with quantification of the substantial risk factors.

## 2. Methods

### 2.1. Patients

We conducted a retrospective review of the medical records of 153 consecutive adult patients with de novo AML undergoing intent-to-cure induction chemotherapy at our hospital between January 2011 and December 2020. Acute promyelocytic leukemia was excluded. Among these 153 patients, 152 received cytarabine (100 mg/m^2^) for 7 days and idarubicin (12 mg/m^2^ for three days; “7 + 3”) or “7 + 3”-like induction chemotherapy. One patient underwent high-dose cytarabine induction. Midostaurin was given to 6 of the 152 patients who received “7 + 3” induction chemotherapy because of their FLT3 mutations. Notably, none of the 153 patients received venetoclax-containing regimens as their remission induction chemotherapy. The disease status of 129 of the 153 patients was assessed after the remission induction chemotherapy. The complete remission rate was 66.7% (86/129). To investigate the potential factors to which early mortality after the remission induction chemotherapy can be attributed, these 153 patients were stratified into the early mortality group (n = 29) and the non-early mortality group (n = 124). This study was approved by the Review Board of Taichung Veterans General Hospital (CE21114A) and was conducted in accordance with the Declaration of Helsinki. The institutional review board agreed to waive the requirement for patients’ informed consent because of the retrospective study design.

### 2.2. Definitions and Outcome Measurements

Early mortality was defined as death by any cause within the first 60 days following remission induction chemotherapy [13]. We described AML as the cause of death if excess blasts remained active at the time of death. The current study compared the difference in clinical characteristics between the early mortality group and the non-early mortality group in terms of outcome measurements. We further investigated the potential factors attributed to early mortality. Age ≥ 65 [14], Eastern Cooperative Oncology Group (ECOG) performance status (PS) ≥ 2 [15], leukocyte count ≥ 100,000 uL [16], estimated glomerular filtration rate (eGFR) < 60 mL/min/1.73 m^2^ [13], alanine aminotransferase (ALT) ≥ 100 U/L [17], lactate dehydrogenase (LDH) ≥ 1000 U/L [18], and poor cytogenetic risks [18] were the risks included as potential factors in the univariate analysis. To focus on rapidly available factors, this analysis did not include genetic profiles as potential risks for AML early mortality. The multivariate analysis analyzed only factors found to be statistically significant in the univariate analysis. The cumulative mortality rates at day 60 were compared according to the various risks associated with early mortality in de novo AML patients undergoing remission induction chemotherapy.

### 2.3. Infection Prophylaxis

Only 1 of the 153 patients received fluoroquinolone prophylaxis during induction, while 47.1% (72/153) received antifungal prophylaxis with posaconazole. No other antifungal prophylaxis was given to patients not receiving posaconazole prophylaxis. Because our previous study demonstrated that posaconazole prophylaxis did not substantially improve overall survival in AML patients [19], we did not investigate the impact of posaconazole prophylaxis on early mortality in the current study.

### 2.4. Statistical Analysis

We used the Mann–Whitney *U* test to compare the continuous variables between the early mortality and non-early mortality groups. Categorical variables were analyzed using the chi-square test or Fisher’s exact test, as required. We used a logistic regression to identify AML early mortality risk factors, quantified as odd ratios (ORs) accompanied with 95% confidence intervals (CIs). Furthermore, we used a log-rank test to compare the cumulative mortality rate at day 60 among the AML patients with different risks and various numbers of risks of early mortality. The results were considered statistically significant when the *p*-value was <0.05.

## 3. Results

### 3.1. Patient Demographics

The median age of the 153 patients in our cohort was 52 (range: 20–73) years. With a median overall survival time of 14.9 months, the mortality rate at day 60 was 19.0% (29/153). With regard to the comparisons of the clinical characteristics among AML patients with and without early mortality, the two groups were comparable in terms of gender (*p =* 0.074), risk of cytogenetics (*p =* 0.255), initial leukocyte count (*p =* 0.798), eGFR (*p =* 0.113), and LDH at diagnosis (*p =* 0.159). However, more patients with early mortality were ≥65 years of age (34.5% vs. 12.1%; *p =* 0.009) and had an ECOG PS ≥ 2 (37.9% vs. 11.3%; *p =* 0.001) than those without early mortality. While patients with early mortality had higher ALT levels, the median ALT levels of the two groups were both within the normal range (30 vs. 23 U/L; *p =* 0.016) (Table 1).

We further investigated the independent factors associated with early mortality of de novo AML patients undergoing remission induction chemotherapy. The univariate analysis revealed that age ≥ 65 years (OR: 3.82; 95% CI: 1.50–9.76; *p =* 0.005), ECOG PS ≥ 2 (OR: 4.80; 95% CI: 1.89–12.22; *p =* 0.001), eGFR < 60 mL/min/1.73 m^2^ (OR: 2.78; 95% CI: 1.04–7.14; *p =* 0.041), and LDH ≥ 1000 IU/L (OR: 3.00; 95% CI: 1.24–7.26; *p =* 0.015) were associated with a higher incidence of AML early mortality. The multivariate analysis further validated this result, showing that age ≥ 65 years (OR: 3.15; 95% CI: 1.05–9.44; *p =* 0.041), ECOG PS ≥ 2 (OR: 4.87; 95% CI: 1.77–13.41; *p =* 0.002), and LDH ≥ 1000 IU/L (OR: 4.20; 95% CI: 1.57–11.23; *p =* 0.004) substantially increased the risks of early mortality in AML patients undergoing remission induction chemotherapy (Table 2).

### 3.2. Patients with Age ≥ 65 Years, ECOG PS ≥ 2, and LDH ≥ 1000 U/L Had Higher Cumulative Early Mortality Rates

**Figure 1:**
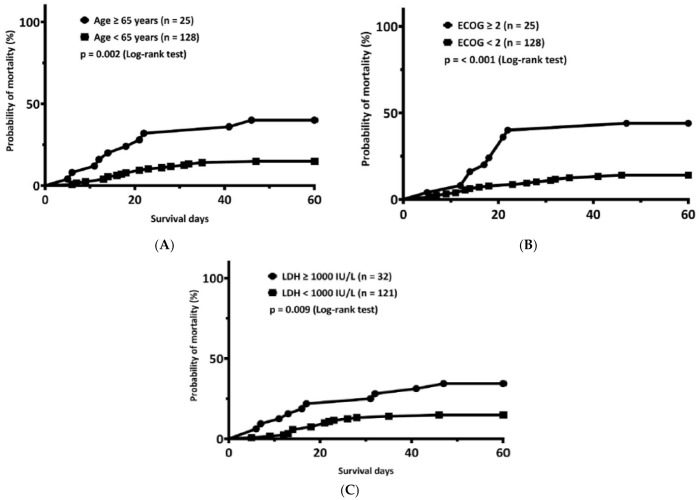
The cumulative incidence of early mortality by risk factors. (**A**) The cumulative early mortality rate of patients ≥65 and <65 years of age was 40.0% and 14.8%, respectively (*p =* 0.002). (**B**) It was 44.0% and 14.1% for patients with Eastern Cooperative Oncology Group (ECOG) performance status (PS) ≥2 and <2 (*p* < 0.001), respectively. (**C**) The cumulative early mortality rate among patients with a lactate dehydrogenase (LDH) ≥1000 and <1000 IU/L was 34.4% and 14.9%, respectively (*p =* 0.009).

Since age ≥ 65 years, ECOG PS ≥ 2, and LDH ≥ 1000 U/L were independent factors associated with early mortality in AML patients undergoing remission induction chemotherapy, we compared the cumulative mortality rates according to these three factors individually. Figure 1 shows the results. The cumulative early mortality rate of patients ≥65 (n = 25) and <65 (n = 128) years of age was 40.0% and 14.8%, respectively (*p =* 0.002, Figure 1A) and 44.0% and 14.1% for patients with an ECOG PS ≥2 (n = 25) and <2 (n = 128), respectively (*p* < 0.001, Figure 1B). Furthermore, the cumulative early mortality rate among patients with LDH ≥1000 (n = 32) and <1000 (n = 121) IU/L was 34.4% and 14.9%, respectively (*p =* 0.009, Figure 1C).

The cumulative mortality rates by day 60 for patients with no risk factors, one risk factor, and two risk factors were 9.2%, 20.0%, and 68.8%, respectively. Patients with two risk factors had a significantly higher early mortality rate than those with one risk factor (*p* < 0.001) and no (*p* < 0.001) risk factors. However, patients with no risk factors and those with one risk factor had a comparable incidence of early mortality (*p =* 0.069).

### 3.3. AML Patients with Two Risk Factors Had a Significantly Higher Early Mortality Rate Than Those with Fewer Than Two Risk Factors

**Figure 2:**
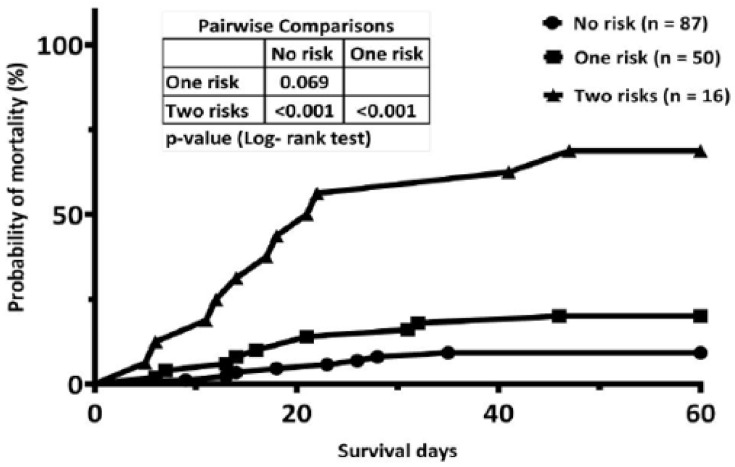
Comparison of the cumulative incidence of early mortality by number of risks.

Next, we examined whether different numbers of risks would impact the probability of early death in AML patients who received remission induction chemotherapy. According to the results of the multivariate analysis, we defined age ≥ 65 years, ECOG PS ≥ 2, and LDH ≥ 1000 IU/L as risk factors for early mortality in AML patients. The cumulative mortality rates by day 60 for patients with no risk factors (n = 87), one risk factor (n = 50), and two risk factors (n = 16) were 9.2%, 20.0%, and 68.8%, respectively. Patients with two risk factors had a significantly higher early mortality rate than those with one (*p* < 0.001) risk factor and no (*p* < 0.001) risk factors. However, patients with no risk factors and those with one risk factor had a comparable incidence of early mortality (*p =* 0.069, Figure 2). There were no patients with three risk factors.

### 3.4. Cause of Death Analysis

The overall mortality rate in this study cohort was 62.1% (95/153). Sepsis remained the most frequent cause of death among patients with early mortality (19/29; 65.5%). However, AML was the leading cause of death among patients who died after day 60 (42/66; 63.6%, Table 3). Notably, intracranial hemorrhage was the cause of death in 10.3% (3/29) of patients with early mortality, suggesting that AML itself or treatment-associated bleeding could be risks for this particular complication during induction chemotherapy.

## 4. Discussion

This study found that the 60-day mortality rate in de novo AML patients undergoing remission induction chemotherapy was 19.0%. Age ≥ 65 years (OR: 3.15; 95% CI: 1.05–9.44; *p =* 0.041), ECOG PS ≥ 2 (OR: 4.87; 95% CI: 1.77–13.41; *p =* 0.002), and LDH ≥ 1000 IU/L (OR: 4.20; 95% CI: 1.57–11.23; *p =* 0.004) were the risk factors that substantially increased early mortality in AML patients. Furthermore, patients with two risk factors had a significantly higher early mortality rate than those with one risk factor (68.8% vs. 20.0%; *p* < 0.001) and those with no risk factors (68.8% vs. 9.2%; *p* < 0.001).

The incidence of early mortality in newly diagnosed AML patients varied in different studies. Heterogeneous definitions of early mortality and patient population were the primary reasons for the data discrepancy. The National Cancer Institute’s Surveillance, Epidemiology, and End Results showed that the incidences of deaths within one and two months after the AML diagnoses were 27% and 38%, respectively [20]. Since 25% of newly diagnosed AML patients are chemotherapy ineligible and receive only the best supportive care, the early mortality rate among AML patients undergoing induction remission therapy may be different from that reported by the National Cancer Institute’s Surveillance, Epidemiology, and End Results. A retrospective analysis of the five Southwest Oncology Group (SWOG) trials showed that the 30-day mortality rate in AML patients receiving induction remission chemotherapy was 12% [14]. In the real-life setting of the current study, the 60-day mortality rate reached 19.0%.

Since approximately 20% of AML patients may die in the first two months after remission induction chemotherapy, the identification of risk factors for early mortality is important. Our study found that age ≥ 65 years, ECOG PS ≥ 2, and LDH ≥ 1000 IU/L were independent factors increasing the risk of early mortality in AML patients who underwent remission induction chemotherapy. Using a combined cohort from SWOG and the MD Anderson Cancer Center, Walter et al. [21] demonstrated a similar result, showing that age was primarily a surrogate that predicted early death after induction therapy for newly diagnosed AML. Furthermore, the findings from additional research [18] have validated our findings in part, by showing that ECOG PS ≥ 2 and higher LDH levels substantially increased the early mortality risk in older patients with AML. However, our analysis did not find that leukocyte count ≥100,000 uL [14], eGFR < 60 mL/min/1.73 m^2^ [13], ALT ≥ 100 U/L [17], and poor cytogenetic risks [18] were attributed to early mortality in AML patients. A small sample size, inconsistent patient characteristics, and various induction regimens may be the explanation for this.

An important finding was that AML patients with at least two of the following risk factors at diagnosis—age ≥ 65 years, ECOG PS ≥ 2, and LDH ≥ 1000 IU/L—may be defined as ineligible for chemotherapy because of a high incidence of early mortality following remission induction chemotherapy. A therapeutic strategy different from “7 + 3” should be considered for such patients. The VIALE-A [22] and VIALE-C [23] trials have demonstrated that venetoclax-based regimens may be one of the choices that can be used in the care of untreated AML patients ≥75 years or those <75 years with significant comorbidities. In addition, 90.9% (10/11) of early mortality in patients with LDH ≥ 1000 IU/L in our study population was due to induction toxicities (data not shown), suggesting that an enormous tumor burden may be associated with more induction death. However, it remains unclear whether venetoclax-based regimens are as effective as conventional remission induction chemotherapy for AML patients with LDH ≥ 1000 IU/L. Nevertheless, using the numbers of particular risks, our research utilized a simple and straightforward approach in the identification of chemotherapy-ineligible, newly diagnosed AML patients, according to the high early mortality incidence.

Notwithstanding these findings, infection remained the leading cause of early death. This result raises the question of the benefit of antimicrobial prophylaxis in AML treatment. A clinical practice guideline proposed by the American Society of Clinical Oncology and Infectious Diseases Society of America recommended fluoroquinolone prophylaxis in AML patients undergoing induction remission chemotherapy, especially those aged ≥ 65 years and with ECOG PS ≥ 2 [24]. However, fluoroquinolone prophylaxis was not part of our routine supportive care approach in AML induction because of the danger of drug resistance [25]. In fact, only 1 of the 153 patients in the current study received fluoroquinolone prophylaxis during remission induction chemotherapy (Supplemental Table S1). Whether antimicrobial prophylaxis can substantially reduce the early mortality of AML patients in a real-life setting remains unknown, and more evidence is required. Nonetheless, it is recommended that patients aged ≥ 65 years with ECOG PS ≥ 2 and LDH ≥ 1000 be the first candidates for fluoroquinolone prophylaxis during their remission induction chemotherapy.

The retrospective study design and the limited number of patients were significant limitations of the current study. Moreover, in order to provide a rapid and simple modality to predict early mortality in de novo AML patients undergoing intention-to-cure induction therapy, we did not include the percentage of myeloblasts in either bone marrow or peripheral blood, AML subtypes and genetic profiles, pre-existing comorbidities, baseline infections, cardiac functions, and antimicrobial prophylaxis in our analyses. Furthermore, an external cohort is required to validate our data. Prospective and randomized controlled studies with large numbers of patients are also required to confirm our results.

In summary, this study showed that early death occurred in 19.0% of newly diagnosed de novo AML patients undergoing remission induction chemotherapy. Older age (≥65 years), poor clinical performance (ECOG PS ≥ 2), and a high tumor burden (LDH ≥ 1000 IU/L) were risks for early mortality. Patients with at least two of these three factors should be more carefully assessed for remission induction chemotherapy. Venetoclax-based regimens might be an alternative approach to reduce premature mortality in these patients.

## Figures and Tables

**Table 1:** Clinical characteristic comparison among patients with and without early mortality.

	All Patients		Without Early Mortality		With Early Mortality		*p*-Value
	(n = 153)		(n = 124)		(n = 29)		
Age, n (%)							0.009 ^a^
<65	128	(83.7%)	109	(87.9%)	19	(65.5%)	
≥65	25	(16.3%)	15	(12.1%)	10	(34.5%)	
Gender, n (%)							0.074 ^b^
Male	91	(59.5%)	69	(55.7%)	22	(75.9%)	
Female	62	(40.5%)	55	(44.3%)	7	(24.1%)	
ECOG performance status, n (%)							0.001 ^a^
<2	128	(83.7%)	110	(88.7%)	18	(62.1%)	
≥2	25	(16.3%)	14	(11.3%)	11	(37.9%)	
Risk of Cytogenetics, n (%)							0.255 ^a^
Unfavorable	32	(20.9%)	25	(20.2%)	7	(24.1%)	
Non-unfavorable	102	(66.7%)	89	(71.8%)	13	(44.8%)	
Undetermined	19	(12.4%)	10	(8.1%)	9	(31.0%)	
Leukocyte (uL), median (range)	41,100	(790–433,220)	40,465	(790–433,220)	41,100	(1000–421,370)	0.798 ^c^
eGFR (ml/min/1.73 m^2^), median (range)	88	(6.6–203.0)	90	(44.0–203.0)	84	(6.6–145.0)	0.113 ^c^
ALT (U/L), median (range)	25	(7–653)	23	(7–653)	30	(9–203)	0.016 ^c^
LDH (U/L), median (range)	563	(146–18,575)	561.5	(155–2919)	761	(146–18,575)	0.159 ^c^

n, number; ECOG, Eastern Cooperative Oncology Group; eGFR, estimated glomerular filtration rate; ALT, alanine aminotransferase; LDH, lactate dehydrogenase. Risks of cytogenetics were determined according to the 2017 European LeukemiaNet classification. Data were compared using ^a^ Fisher’s exact test, ^b^ chi-square test, and ^c^ Mann–Whitney U test.

**Table 2:** Risk factors associated with early mortality.

	Univariate Analysis			Multivariate Analysis		
	OR	95% CI	*p*-Value	OR	95% CI	*p*-Value
Age ≥ 65	3.82	(1.50–9.76)	0.005	3.15	(1.05–9.44)	0.041
ECOG performance status ≥ 2	4.80	(1.89–12.22)	0.001	4.87	(1.77–13.41)	0.002
Leukocyte count ≥ 100,000 uL	1.09	(0.42–2.82)	0.857			
eGFR < 60 mL/min/1.73 m^2^	2.78	(1.04–7.14)	0.041	0.63	(0.20–1.98)	0.433
ALT ≥ 100 U/L	0.60	(0.07–5.05)	0.636			
LDH ≥ 1000 U/L	3.00	(1.24–7.26)	0.015	4.20	(1.57–11.23)	0.004
Poor cytogenetic risks	1.92	(0.69–5.32)	0.211			

ECOG PS, Eastern Cooperative Oncology Group; eGFR, estimated glomerular filtration rate; ALT, alanine aminotransferase; LDH, lactate dehydrogenase; OR, odds ratio; CI, confidence interval.

**Table 3:** Causes of death.

	Patients with Mortality		With Early Mortality		Without Early Mortality	
	(n = 95)		(n = 29)		(n = 66)	
	n	(%)	n	(%)	n	(%)
Acute myeloid leukemia	46	48.4	4	13.8	42	63.6
Sepsis	32	33.7	19	65.5	13	19.7
Graft-versus-host disease	6	6.3	0	0.0	6	9.1
Pneumonia	5	5.3	2	6.9	3	4.6
Intracranial hemorrhage	4	4.2	3	10.3	1	1.5
Cytomegalovirus infection	1	1.1	0	0.0	1	1.5
Gastrointestinal bleeding	1	1.1	1	3.5	0	0.0

## Data Availability

Data are available upon reasonable request.

## Supplementary Materials

The following are available online at https://www.mdpi.com/article/10.3390/jcm10245768/s1, Table S1: Clinical characteristic of the patient using prophylactic fluoroquinolone.
