# Supplementary material for: Redefining Remission Induction Chemotherapy Ineligibility by Early Mortality in De Novo Acute Myeloid Leukemia
Source: J Clin Med. 2021 Dec 9;10(24):5768. doi: 10.3390/jcm10245768 (PMC8708870; doi:10.3390/jcm10245768)
Supplement: Supplementary file 1 [file jcm-10-05768-s001.zip › jcm-1429046-supplementary.pdf]

Supplemental Table S1. Clinical characteristic of the patient using prophylactic fluoroquinolone

Age	Gender	LDH	ECOG PS	Dose	Survival days	Cause of death
53	Male	271	0	Daily levofloxacin (500mg) for 5 days during the nadir	313	Graft versus host disease

LDH: lactate dehydrogenase; ECOG: Eastern Cooperative Oncology Group; PS: perfornance status
